# 2-(Benzo[*d*]thia­zol-2-ylsulfon­yl)-1-(4-bromo­phen­yl)ethanone

**DOI:** 10.1107/S1600536809053112

**Published:** 2009-12-19

**Authors:** Hossein Loghmani-Khouzani, Dariush Hajiheidari, Ward T. Robinson, Reza Kia

**Affiliations:** a Chemistry Department, University of Isfahan, Isfahan, 81746-73441, Iran; b University of Malaya, Department of Chemistry, 50603, Kuala Lumpur, Malaysia; c Department of Chemistry, Science and Research Campus, Islamic Azad University, Poonak, Tehran, Iran

## Abstract

In the title mol­ecule, C_15_H_10_BrNO_3_S_2_, the dihedral angle between the benzothia­zole ring system and the benzene ring is 67.57 (12)°. The crystal structure is stabilized by weak inter­molecular C—H⋯O inter­actions. In addition, there is an inter­molecular Br⋯C [3.379 (3) Å] contact which is shorter than the sum of the van der Waals radii of these atoms.

## Related literature

For bond-length data, see Allen *et al.* (1987 ▶). For the applications of related compounds in organic synthesis, see: Marco *et al.* (1995 ▶); Fuju *et al.* (1988 ▶); Ni *et al.* (2006 ▶); Grossert *et al.* (1984 ▶); Oishi *et al.* (1988) ▶; Antane *et al.* (2004 ▶). For the biological activity of related compounds see, Padmavathi *et al.* (2008 ▶). For related structures see: Loghmani-Khouzani *et al.* (2008 ▶, 2009*a* ▶,*b* ▶); Munoz *et al.* (2005 ▶); Suryakiran *et al.* (2007 ▶).
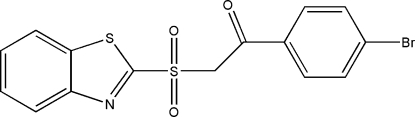


## Experimental

### 

#### Crystal data

C_15_H_10_BrNO_3_S_2_
*M* *_r_* = 396.27
Monoclinic, 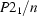

*a* = 5.6695 (10) Å
*b* = 24.489 (4) Å
*c* = 10.7042 (19) Å
β = 94.178 (3)°
*V* = 1482.2 (5) Å^3^
*Z* = 4
Mo *K*α radiation
μ = 3.07 mm^−1^
*T* = 296 K
0.42 × 0.30 × 0.05 mm

#### Data collection

Bruker SMART APEXII CCD area-detector diffractometer
Absorption correction: multi-scan (*SADABS*; Bruker, 2005 ▶) *T*_min_ = 0.363, *T*_max_ = 0.864
6602 measured reflections
2564 independent reflections
2140 reflections with *I* > 2σ(*I*)
*R*_int_ = 0.038

#### Refinement

*R*[*F* ^2^ > 2σ(*F* ^2^)] = 0.036
*wR*(*F* ^2^) = 0.089
*S* = 1.03
2564 reflections
199 parameters
H-atom parameters constrained
Δρ_max_ = 0.65 e Å^−3^
Δρ_min_ = −0.84 e Å^−3^

### 

Data collection: *APEX2* (Bruker, 2005 ▶); cell refinement: *SAINT* (Bruker, 2005 ▶); data reduction: *SAINT*; program(s) used to solve structure: *SHELXTL* (Sheldrick, 2008 ▶); program(s) used to refine structure: *SHELXTL*; molecular graphics: *SHELXTL*; software used to prepare material for publication: *SHELXTL* and *PLATON* (Spek, 2009 ▶).

## Supplementary Material

Crystal structure: contains datablocks global, I. DOI: 10.1107/S1600536809053112/lh2971sup1.cif

Structure factors: contains datablocks I. DOI: 10.1107/S1600536809053112/lh2971Isup2.hkl

Additional supplementary materials:  crystallographic information; 3D view; checkCIF report

## Figures and Tables

**Table 1:** Hydrogen-bond geometry (Å, °)

*D*—H⋯*A*	*D*—H	H⋯*A*	*D*⋯*A*	*D*—H⋯*A*
C4—H4*A*⋯O2^i^	0.93	2.56	3.420 (4)	154
C8—H8*A*⋯O1^ii^	0.97	2.37	3.289 (3)	158
C8—H8*B*⋯O1^iii^	0.97	2.50	3.241 (4)	133
C14—H14*A*⋯O2^iv^	0.93	2.56	3.226 (4)	128

Symmetry codes: (i) 
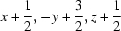
; (ii) 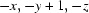; (iii) 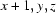; (iv) 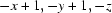.

**Fig. 1.**
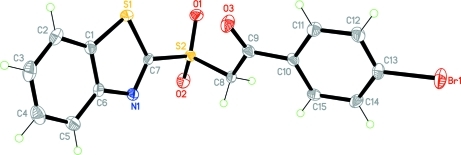
The molecular structure of the title compound, showing 50% probability displacement ellipsoids and the atomic numbering.

**Fig. 2.**
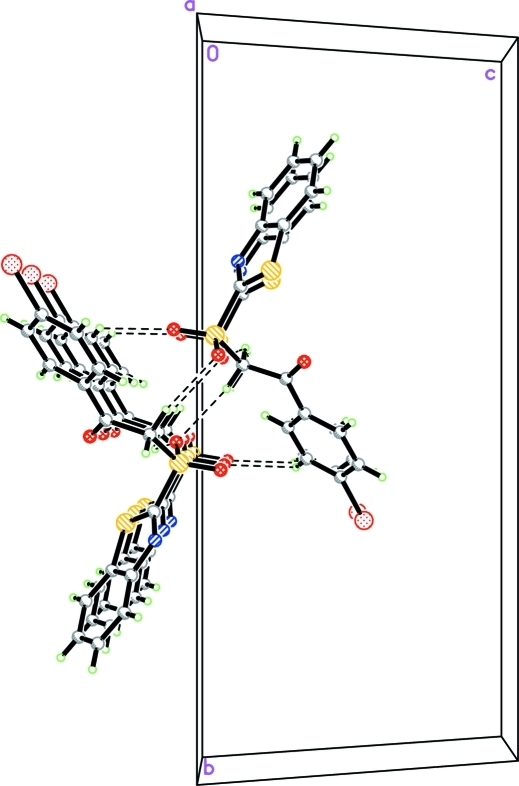
Part of the crystal structure of the title compound, viewed approximately along the a-axis, showing molecules connected via weak intermolecular C—H···O interactions. Intermolecular interactions are drawn as dashed lines.

## supplementary crystallographic information

### Comment

The existence of so many valence states of sulfur has generated selective and novel ways to affect oxidation, dehydration, and carbon-carbon bond formation (Loghmani-Khouzani *et al.,* 2008). Recent methods that allow introduction of a sulfur constituent β to a carbonyl group have shown particular promise (Loghmani-Khouzani *et al.,* 2009*a*,*b*; Suryakiran *et al.,* 2007; Munoz *et al.,* 2005). 2-(1,3-Benzothiazol-2-yl-sulfonyl)-1-(4-bromophenyl)ethanone as a new compound with sulfur atom β to the carbonyl group is of great importance in organic synthesis. β-Keto-sulfones are a very important group of intermediates as they are precursors for Michael and Knoevenagel reactions (Marco *et al.,* 1995) and are used in the preparation of acetylenes, allenes, chalcones, vinyl sulfones, polyfunctionalized 4H-pyrans and ketones (Fuju *et al.,* 1988). In addition, β-keto-sulfones can be converted into optically active β-hydroxy-sulfones, halomethyl sulfones and dihalomethyl sulfones (Ni *et al.,* 2006). Halomethyl sulfones and dihalomethyl sulfones are very good α-carbanion stabilizing substituents and precursors for the preparation of alkenes, aziridines, epoxides, and β-hydroxy-sulfones (Grossert *et al.,* 1984). Haloalkyl sulfones are useful in preventing aquatic organisms from attaching to fishing nets and ship hulls (Oishi *et al.,* 1988). They also possess other biological properties such as herbicidal, bactericidal antifungal, algaecidal and insecticidal (Antane *et al.,* 2004). Recently sulfone-linked heterocycles were prepared and have been showed antimicrobial activity (Padmavathi *et al.,* 2008). We report here the crystal structure of the title compound as a precursor for synthesis of gem-difluoromethylene-containing heterocycle.

In the molecule of the title compound, (Fig. 1), a new thio-benzothiazole derivative, the bond lengths (Allen *et al.,* 1987) and angles are within the normal values and are comparable to the related structures (Loghmani-Khouzani *et al.,* 2008*a*,*b*). The dihedral angle between the benzothiazole ring system and the  benzene ring is 67.57 (12)°. An interesting feature of the crystal structure is the short intermolecular Br···C^iv^ [3.379 (3) Å; (iv) -x, -y, 2 - z] contact which is shorter than the sum of the van der Waals radii of these atoms. The crystal structure is stabilized by weak intermolecular C—H···O interactions (Table 1, Fig. 2).

### Experimental

Sodium carbonate (4.5 mmol) was added to a stirred solution of 2-mercaptobenzothiazol (3 mmol) in ethanol (15 mL) and water (15 mL) and stirred at room temperature for 30 min. 2-bromo-1- (4-bromophenyl)ethanone (3 mmol) was added to the reaction mixture and stirring was continued for 1 h. The reaction was monitored by TLC and after 60 min. showed the complete disappearance of starting materials. The reaction mixture was poured into 100 mL of 1 M HCl containing 50 g of crushed ice. The product was filtered under vacuum and the filtrate was washed with 10 mL ice-cold ethanol and 10 mL water. Recrystallization from petroleum ether and filtration gave 2-(Benzo[d]thiazol-2-ylthio)-1-(4-bromophenyl)ethanone. The product yield was 96%. For oxidation of the resulting Product, *m*-CPBA (3 mmol) was added to a solution of 2-(1,3-Benzothiazol-2- yl-thio)-1-(4-bromophenyl)ethanone  (1 mmol) in CH_2_Cl_2_ (20 mL) under stirring at 273K. The mixture was stirred at room temperature for 1 h to complete the reaction. Saturated aqueous sodium sulfite solution (50 mL) was added and the mixture was stirred for a further 1 h at room temperature. The CH_2_Cl_2_ layer was washed with water (50 mL), dried (MgSO_4_), filtered, and concentrated under reduced pressure. Flash chromatography on silica gel using AcOEt/petroleum ether (30:70) afforded 2-(1,3-Benzothiazol-2-ylsulfonyl)-1-(4-bromophenyl) ethanone. The product yield of the resulted β-ketosulfone was 80 %. White solid; m.p.: 196-198 ^°^C; ^1^H-NMR (400 MHz; CDCl_3_): δ 8.16-7.47 (m, 8H), 5.67 (s, 2H). ^13^C-NMR (126 MHz; CDCl_3_): δ 188.1 (C=O), 154.1, 151.5, 134.8, 133.2, 129.7, 129.1, 126.2, 123.9, 123.1, 121.7, 121.2, 59.8. IR (KBr, cm^-1^ ): 3010, 2800, 1684 (C=O), 1570, 1401, 1328, 1150, 1122, 970, 803, 752. Anal. Calcd for C_15_H_10_BrNO_3_S_2_: C, 45.46; H, 2.54; N, 3.53. Found: C, 45.49; H, 2.50; N, 3.43.

### Refinement

All of the hydrogen atoms were positioned geometrically [C—H = 0.93–0.97 Å] and refined using a riding model approximation with U_iso_ (H) = 1.2 U_eq_ (C).

### Crystal data

**Table:**

C_15_H_10_BrNO_3_S_2_	*F*(000) = 792
*M**_r_* = 396.27	*D*_x_ = 1.776 Mg m^−^^3^
Monoclinic, *P*2_1_/*n*	Melting point: 470 K
Hall symbol: -P 2yn	Mo *K*α radiation, λ = 0.71073 Å
*a* = 5.6695 (10) Å	Cell parameters from 3475 reflections
*b* = 24.489 (4) Å	θ = 2.5–30.3°
*c* = 10.7042 (19) Å	µ = 3.07 mm^−^^1^
β = 94.178 (3)°	*T* = 296 K
*V* = 1482.2 (5) Å^3^	Plate, colourless
*Z* = 4	0.42 × 0.30 × 0.05 mm

### Data collection

**Table:**

Bruker SMART APEXII CCD area-detector diffractometer	2564 independent reflections
Radiation source: fine-focus sealed tube	2140 reflections with *I* > 2σ(*I*)
graphite	*R*_int_ = 0.038
φ and ω scans	θ_max_ = 25.0°, θ_min_ = 2.5°
Absorption correction: multi-scan (*SADABS*; Bruker, 2005)	*h* = −6→6
*T*_min_ = 0.363, *T*_max_ = 0.864	*k* = −29→23
6602 measured reflections	*l* = −12→12

### Refinement

**Table:**

Refinement on *F*^2^	Primary atom site location: structure-invariant direct methods
Least-squares matrix: full	Secondary atom site location: difference Fourier map
*R*[*F*^2^ > 2σ(*F*^2^)] = 0.036	Hydrogen site location: inferred from neighbouring sites
*wR*(*F*^2^) = 0.089	H-atom parameters constrained
*S* = 1.03	*w* = 1/[σ^2^(*F*_o_^2^) + (0.0547*P*)^2^] where *P* = (*F*_o_^2^ + 2*F*_c_^2^)/3
2564 reflections	(Δ/σ)_max_ = 0.001
199 parameters	Δρ_max_ = 0.65 e Å^−^^3^
0 restraints	Δρ_min_ = −0.83 e Å^−^^3^

### Special details

**Table:**

Geometry. All esds (except the esd in the dihedral angle between two l.s. planes) are estimated using the full covariance matrix. The cell esds are taken into account individually in the estimation of esds in distances, angles and torsion angles; correlations between esds in cell parameters are only used when they are defined by crystal symmetry. An approximate (isotropic) treatment of cell esds is used for estimating esds involving l.s. planes.
Refinement. Refinement of F^2^ against ALL reflections. The weighted R-factor wR and goodness of fit S are based on F^2^, conventional R-factors R are based on F, with F set to zero for negative F^2^. The threshold expression of F^2^ > 2sigma(F^2^) is used only for calculating R-factors(gt) etc. and is not relevant to the choice of reflections for refinement. R-factors based on F^2^ are statistically about twice as large as those based on F, and R- factors based on ALL data will be even larger.

### Fractional atomic coordinates and isotropic or equivalent isotropic displacement parameters (Å^2^)

**Table:**

	*x*	*y*	*z*	*U*_iso_*/*U*_eq_	
Br1	0.73768 (6)	0.334681 (12)	0.50902 (3)	0.02981 (14)	
C6	0.1307 (5)	0.72648 (11)	0.1923 (3)	0.0176 (6)	
C5	0.2796 (6)	0.77145 (12)	0.2066 (3)	0.0254 (7)	
H5A	0.4151	0.7739	0.1630	0.031*	
C4	0.2209 (6)	0.81234 (12)	0.2872 (3)	0.0285 (8)	
H4A	0.3187	0.8427	0.2984	0.034*	
C3	0.0172 (6)	0.80883 (13)	0.3521 (3)	0.0278 (8)	
H3A	−0.0167	0.8368	0.4069	0.033*	
C2	−0.1355 (6)	0.76516 (12)	0.3374 (3)	0.0234 (7)	
H2A	−0.2719	0.7633	0.3805	0.028*	
C1	−0.0776 (5)	0.72389 (11)	0.2555 (3)	0.0180 (6)	
C7	0.0046 (5)	0.64645 (11)	0.1287 (3)	0.0152 (6)	
C8	0.2422 (5)	0.54542 (11)	0.1342 (3)	0.0161 (6)	
H8A	0.2775	0.5135	0.0852	0.019*	
H8B	0.3830	0.5680	0.1414	0.019*	
C9	0.1874 (5)	0.52687 (11)	0.2645 (3)	0.0187 (7)	
C10	0.3307 (5)	0.48088 (11)	0.3204 (3)	0.0165 (6)	
C15	0.5345 (5)	0.46196 (11)	0.2724 (3)	0.0194 (7)	
H15A	0.5888	0.4782	0.2013	0.023*	
C14	0.6590 (5)	0.41889 (11)	0.3296 (3)	0.0208 (7)	
H14A	0.7975	0.4063	0.2979	0.025*	
C13	0.5753 (5)	0.39509 (11)	0.4333 (3)	0.0202 (7)	
C12	0.3738 (6)	0.41345 (11)	0.4839 (3)	0.0225 (7)	
H12A	0.3209	0.3971	0.5551	0.027*	
C11	0.2519 (6)	0.45649 (12)	0.4270 (3)	0.0226 (7)	
H11A	0.1154	0.4694	0.4603	0.027*	
N1	0.1732 (4)	0.68070 (9)	0.1198 (2)	0.0176 (6)	
O2	0.0855 (4)	0.59122 (8)	−0.07094 (18)	0.0240 (5)	
O1	−0.2124 (4)	0.55675 (8)	0.0658 (2)	0.0225 (5)	
O3	0.0318 (4)	0.54839 (9)	0.3174 (2)	0.0296 (6)	
S2	0.01146 (13)	0.58258 (3)	0.05223 (7)	0.01619 (19)	
S1	−0.22406 (13)	0.66352 (3)	0.21941 (7)	0.0196 (2)	

### Atomic displacement parameters (Å^2^)

**Table:**

	*U*^11^	*U*^22^	*U*^33^	*U*^12^	*U*^13^	*U*^23^
Br1	0.0356 (2)	0.0266 (2)	0.0268 (2)	0.01244 (13)	−0.00100 (16)	0.00696 (13)
C6	0.0204 (16)	0.0135 (14)	0.0185 (15)	0.0026 (12)	−0.0011 (13)	0.0023 (11)
C5	0.0218 (17)	0.0234 (16)	0.0309 (18)	−0.0031 (13)	0.0000 (14)	0.0022 (14)
C4	0.034 (2)	0.0193 (16)	0.0311 (19)	−0.0034 (14)	−0.0076 (16)	−0.0036 (14)
C3	0.035 (2)	0.0209 (16)	0.0269 (18)	0.0068 (14)	−0.0024 (16)	−0.0078 (13)
C2	0.0246 (17)	0.0228 (16)	0.0227 (17)	0.0080 (13)	0.0014 (14)	−0.0007 (12)
C1	0.0195 (16)	0.0144 (13)	0.0199 (16)	0.0022 (12)	−0.0008 (13)	0.0027 (12)
C7	0.0165 (15)	0.0131 (14)	0.0159 (16)	0.0031 (11)	0.0013 (13)	0.0010 (11)
C8	0.0169 (15)	0.0120 (13)	0.0196 (16)	0.0020 (11)	0.0029 (13)	0.0013 (11)
C9	0.0219 (16)	0.0167 (14)	0.0180 (16)	−0.0032 (13)	0.0045 (14)	−0.0015 (12)
C10	0.0199 (16)	0.0140 (13)	0.0161 (15)	−0.0011 (12)	0.0052 (13)	−0.0004 (11)
C15	0.0198 (16)	0.0226 (15)	0.0168 (16)	−0.0014 (12)	0.0085 (13)	0.0031 (12)
C14	0.0176 (15)	0.0248 (16)	0.0210 (16)	0.0048 (12)	0.0078 (13)	−0.0007 (13)
C13	0.0275 (17)	0.0164 (14)	0.0161 (16)	0.0040 (13)	−0.0015 (14)	0.0013 (12)
C12	0.0281 (18)	0.0223 (15)	0.0181 (16)	0.0040 (14)	0.0100 (14)	0.0070 (12)
C11	0.0234 (17)	0.0271 (16)	0.0184 (16)	0.0059 (13)	0.0080 (14)	0.0003 (13)
N1	0.0183 (14)	0.0164 (12)	0.0184 (13)	0.0011 (10)	0.0044 (11)	0.0023 (10)
O2	0.0311 (12)	0.0264 (11)	0.0148 (11)	0.0048 (9)	0.0039 (10)	0.0014 (8)
O1	0.0169 (11)	0.0198 (10)	0.0310 (13)	−0.0034 (9)	0.0028 (10)	−0.0038 (9)
O3	0.0376 (14)	0.0288 (12)	0.0244 (12)	0.0144 (10)	0.0170 (11)	0.0054 (10)
S2	0.0176 (4)	0.0148 (4)	0.0163 (4)	0.0011 (3)	0.0025 (3)	−0.0004 (3)
S1	0.0183 (4)	0.0182 (4)	0.0230 (4)	−0.0002 (3)	0.0072 (3)	0.0000 (3)

### Geometric parameters (Å, °)

**Table:**

Br1—C13	1.893 (3)	C8—S2	1.773 (3)
C6—C5	1.389 (4)	C8—H8A	0.9700
C6—N1	1.395 (4)	C8—H8B	0.9700
C6—C1	1.404 (4)	C9—O3	1.204 (4)
C5—C4	1.378 (5)	C9—C10	1.489 (4)
C5—H5A	0.9300	C10—C15	1.378 (4)
C4—C3	1.393 (5)	C10—C11	1.391 (4)
C4—H4A	0.9300	C15—C14	1.386 (4)
C3—C2	1.378 (4)	C15—H15A	0.9300
C3—H3A	0.9300	C14—C13	1.369 (4)
C2—C1	1.393 (4)	C14—H14A	0.9300
C2—H2A	0.9300	C13—C12	1.375 (4)
C1—S1	1.726 (3)	C12—C11	1.378 (4)
C7—N1	1.281 (4)	C12—H12A	0.9300
C7—S1	1.726 (3)	C11—H11A	0.9300
C7—S2	1.767 (3)	O2—S2	1.428 (2)
C8—C9	1.520 (4)	O1—S2	1.435 (2)
			
C5—C6—N1	124.8 (3)	O3—C9—C8	120.5 (3)
C5—C6—C1	120.5 (3)	C10—C9—C8	116.9 (3)
N1—C6—C1	114.6 (2)	C15—C10—C11	119.3 (3)
C4—C5—C6	118.2 (3)	C15—C10—C9	123.5 (3)
C4—C5—H5A	120.9	C11—C10—C9	117.1 (3)
C6—C5—H5A	120.9	C10—C15—C14	120.3 (3)
C5—C4—C3	120.9 (3)	C10—C15—H15A	119.8
C5—C4—H4A	119.5	C14—C15—H15A	119.8
C3—C4—H4A	119.5	C13—C14—C15	119.1 (3)
C2—C3—C4	121.8 (3)	C13—C14—H14A	120.5
C2—C3—H3A	119.1	C15—C14—H14A	120.5
C4—C3—H3A	119.1	C14—C13—C12	121.8 (3)
C3—C2—C1	117.4 (3)	C14—C13—Br1	119.6 (2)
C3—C2—H2A	121.3	C12—C13—Br1	118.6 (2)
C1—C2—H2A	121.3	C13—C12—C11	118.7 (3)
C2—C1—C6	121.0 (3)	C13—C12—H12A	120.6
C2—C1—S1	129.1 (2)	C11—C12—H12A	120.6
C6—C1—S1	109.8 (2)	C12—C11—C10	120.7 (3)
N1—C7—S1	118.6 (2)	C12—C11—H11A	119.7
N1—C7—S2	120.2 (2)	C10—C11—H11A	119.7
S1—C7—S2	121.18 (17)	C7—N1—C6	109.0 (3)
C9—C8—S2	114.5 (2)	O2—S2—O1	118.72 (13)
C9—C8—H8A	108.6	O2—S2—C7	108.34 (13)
S2—C8—H8A	108.6	O1—S2—C7	107.07 (13)
C9—C8—H8B	108.6	O2—S2—C8	106.09 (13)
S2—C8—H8B	108.6	O1—S2—C8	110.47 (13)
H8A—C8—H8B	107.6	C7—S2—C8	105.39 (13)
O3—C9—C10	122.6 (3)	C1—S1—C7	87.97 (14)
			
N1—C6—C5—C4	−176.3 (3)	C14—C13—C12—C11	1.0 (5)
C1—C6—C5—C4	2.1 (4)	Br1—C13—C12—C11	−178.3 (2)
C6—C5—C4—C3	−0.4 (4)	C13—C12—C11—C10	0.1 (5)
C5—C4—C3—C2	−1.0 (5)	C15—C10—C11—C12	−0.7 (4)
C4—C3—C2—C1	0.6 (4)	C9—C10—C11—C12	179.6 (3)
C3—C2—C1—C6	1.1 (4)	S1—C7—N1—C6	1.1 (3)
C3—C2—C1—S1	178.0 (2)	S2—C7—N1—C6	−177.54 (19)
C5—C6—C1—C2	−2.5 (4)	C5—C6—N1—C7	178.7 (3)
N1—C6—C1—C2	176.0 (3)	C1—C6—N1—C7	0.3 (3)
C5—C6—C1—S1	−180.0 (2)	N1—C7—S2—O2	−43.5 (3)
N1—C6—C1—S1	−1.4 (3)	S1—C7—S2—O2	137.99 (16)
S2—C8—C9—O3	−19.1 (4)	N1—C7—S2—O1	−172.6 (2)
S2—C8—C9—C10	159.8 (2)	S1—C7—S2—O1	8.8 (2)
O3—C9—C10—C15	−168.3 (3)	N1—C7—S2—C8	69.7 (3)
C8—C9—C10—C15	12.9 (4)	S1—C7—S2—C8	−108.81 (18)
O3—C9—C10—C11	11.5 (4)	C9—C8—S2—O2	−174.09 (19)
C8—C9—C10—C11	−167.4 (3)	C9—C8—S2—O1	−44.2 (2)
C11—C10—C15—C14	0.3 (4)	C9—C8—S2—C7	71.1 (2)
C9—C10—C15—C14	180.0 (3)	C2—C1—S1—C7	−175.6 (3)
C10—C15—C14—C13	0.7 (4)	C6—C1—S1—C7	1.6 (2)
C15—C14—C13—C12	−1.4 (5)	N1—C7—S1—C1	−1.6 (2)
C15—C14—C13—Br1	177.9 (2)	S2—C7—S1—C1	176.96 (18)

### Hydrogen-bond geometry (Å, °)

**Table:**

*D*—H···*A*	*D*—H	H···*A*	*D*···*A*	*D*—H···*A*
C4—H4A···O2^i^	0.93	2.56	3.420 (4)	154
C8—H8A···O1^ii^	0.97	2.37	3.289 (3)	158
C8—H8B···O1^iii^	0.97	2.50	3.241 (4)	133
C14—H14A···O2^iv^	0.93	2.56	3.226 (4)	128

Symmetry codes: (i) *x*+1/2, −*y*+3/2, *z*+1/2; (ii) −*x*, −*y*+1, −*z*; (iii) *x*+1, *y*, *z*; (iv) −*x*+1, −*y*+1, −*z*.
